# Supplementary material for: Head and Neck Cutaneous Soft-Tissue Sarcoma Demonstrate Sex and Racial/Ethnic Disparities in Incidence and Socioeconomic Disparities in Survival
Source: J Clin Med. 2022 Sep 17;11(18):5475. doi: 10.3390/jcm11185475 (PMC9501210; doi:10.3390/jcm11185475)
Supplement: Supplementary file 1 [file jcm-11-05475-s001.zip › jcm-1903392-supplementary.pdf]

Histology recode - broad groupings

		Frequency	Percent	Valid Percent	Cumulative Percent
Valid	8800-8809: soft tissue tumors an	673	15.8	15.8	15.8
	8810-8839: fibromatous neoplasms	2738	64.4	64.4	80.2
	8840-8849: myxomatous neoplasms	1	0.0	0.0	80.2
	8850-8889: lipomatous neoplasms	13	0.3	0.3	80.5
	8890-8929: myomatous neoplasms	296	7.0	7.0	87.5
	8930-8999: complex mixed and str	17	0.4	0.4	87.9
	9120-9169: blood vessel tumors	515	12.1	12.1	100.0
	Total	4253	100.0	100.0	

ICD-O-3 Hist/behav, malignant

		Frequency	Percent	Valid Percent	Cumulative Percent
Valid	8800/3: Sarcoma, NOS	93	2.2	2.2	2.2
	8801/3: Spindle cell sarcoma	62	1.5	1.5	3.6
	8802/3: Giant cell sarcoma	461	10.8	10.8	14.5
	8803/3: Small cell sarcoma	1	0.0	0.0	14.5
	8804/3: Epithelioid sarcoma	10	0.2	0.2	14.7
	8805/3: Undifferentiated sarcoma	45	1.1	1.1	15.8
	8806/3: Desmoplastic small round cell	1	0.0	0.0	15.8
	8810/3: Fibrosarcoma, NOS	13	0.3	0.3	16.1
	8811/3: Fibromyxosarcoma	8	0.2	0.2	16.3
	8815/3: Solitary fibrous tumor, malign	3	0.1	0.1	16.4

8825/3: Myofibroblastoma, malignant	1	0.0	0.0	16.4
8830/3: Malignant fibrous histiocytoma	2026	47.6	47.6	64.0
8832/3: Dermatofibrosarcoma, NOS	672	15.8	15.8	79.8
8833/3: Pigmented dermatofibrosarcoma	13	0.3	0.3	80.2
8836/3: Malignant angiomatoid fibrous	2	0.0	0.0	80.2
8840/3: Myxosarcoma	1	0.0	0.0	80.2
8850/3: Liposarcoma, NOS	1	0.0	0.0	80.2
8851/3: Liposarcoma, well differentiat	2	0.0	0.0	80.3
8852/3: Myxoid liposarcoma	2	0.0	0.0	80.3
8854/3: Pleomorphic liposarcoma	7	0.2	0.2	80.5
8858/3: Dedifferentiated liposarcoma	1	0.0	0.0	80.5
8890/3: Leiomyosarcoma, NOS	276	6.5	6.5	87.0
8891/3: Epithelioid leiomyosarcoma	12	0.3	0.3	87.3
8894/3: Angiomyosarcoma	3	0.1	0.1	87.4
8896/3: Myxoid leiomyosarcoma	1	0.0	0.0	87.4
8900/3: Rhabdomyosarcoma, NOS	1	0.0	0.0	87.4
8901/3: Pleomorphic rhabdomyosarcoma,	1	0.0	0.0	87.4
8910/3: Embryonal rhabdomyosarcoma, NO	1	0.0	0.0	87.5
8920/3: Alveolar rhabdomyosarcoma	1	0.0	0.0	87.5
8935/3: Stromal sarcoma, NOS	17	0.4	0.4	87.9
9120/3: Hemangiosarcoma	504	11.9	11.9	99.7

9130/3: Hemangioendothelioma, malignan	6	0.1	0.1	99.9
9133/3: Epithelioid hemangioendothelio	5	0.1	0.1	100.0
Total	4253	100.0	100.0	
